# Facial Genetics: A Brief Overview

**DOI:** 10.3389/fgene.2018.00462

**Published:** 2018-10-16

**Authors:** Stephen Richmond, Laurence J. Howe, Sarah Lewis, Evie Stergiakouli, Alexei Zhurov

**Affiliations:** ^1^Applied Clinical Research and Public Health, School of Dentistry, College of Biomedical and Life Sciences, Cardiff University, Cardiff, United Kingdom; ^2^MRC Integrative Epidemiology Unit, Population Health Sciences, University of Bristol, Bristol, United Kingdom; ^3^Institute of Cardiovascular Science, University College London, London, United Kingdom; ^4^School of Oral and Dental Sciences, University of Bristol, Bristol, United Kingdom

**Keywords:** 3D imaging, admixture, ancestry, facial variation, geometric morphometrics, facial genetics, facial phenotyping, genetic-environmental influences

## Abstract

Historically, craniofacial genetic research has understandably focused on identifying the causes of craniofacial anomalies and it has only been within the last 10 years, that there has been a drive to detail the biological basis of normal-range facial variation. This initiative has been facilitated by the availability of low-cost hi-resolution three-dimensional systems which have the ability to capture the facial details of thousands of individuals quickly and accurately. Simultaneous advances in genotyping technology have enabled the exploration of genetic influences on facial phenotypes, both in the present day and across human history.

There are several important reasons for exploring the genetics of normal-range variation in facial morphology.

- Disentangling the environmental factors and relative parental biological contributions to heritable traits can help to answer the age-old question “why we look the way that we do?”

- Understanding the etiology of craniofacial anomalies; e.g., unaffected family members of individuals with non-syndromic cleft lip/palate (nsCL/P) have been shown to differ in terms of normal-range facial variation to the general population suggesting an etiological link between facial morphology and nsCL/P.

- Many factors such as ancestry, sex, eye/hair color as well as distinctive facial features (such as, shape of the chin, cheeks, eyes, forehead, lips, and nose) can be identified or estimated using an individual’s genetic data, with potential applications in healthcare and forensics.

- Improved understanding of historical selection and adaptation relating to facial phenotypes, for example, skin pigmentation and geographical latitude.

- Highlighting what is known about shared facial traits, medical conditions and genes.

## Introduction

The facial surface is readily visible and identifiable with a close relationship to the underlying cartilaginous and skeletal structures (Stephan et al., 2005; Wilkinson et al., 2006; De Greef et al., 2006; Al Ali et al., 2014b; Shrimpton et al., 2014). Differences in relative size, shape and spatial arrangement (vertical, horizontal and depth) between the various facial features (e.g., eyes, nose, lips etc.) make each individual human face unique, although closely related individuals such as monozygotic twins have very similar facial structures. Information on an individual’s facial morphology can have several important clinical and forensic applications; informing patient specific models, improving and reducing the need for extensive surgical interventions for craniofacial anomalies/trauma, prediction/reconstruction of the facial form from skeletal remains, and identification of suspects from DNA (Stephan et al., 2005; De Greef et al., 2006; Wilkinson et al., 2006; Beldie et al., 2010; Popat et al., 2010, 2012; Richmond et al., 2012; Al Ali et al., 2014a; Shrimpton et al., 2014; Farnell et al., 2017; Richmond S. et al., 2018).

## Pre- and Post-Natal Facial Development

The development of the face involves a coordinated complex series of embryonic events. Recognizable features of the human face develop around the 4th week of gestation and are closely related to cranial neural crest cells (Marcucio et al., 2015). The facial developmental component processes are listed (**Table 1**) and the human embryonic sequence of events can be visualized which aids understanding of the movement of the facial processes followed by their fusion (Sharman, 2011).

**Table 1:** Embryonic features that contribute to facial development.

Developmental facial processes	Facial features
Frontonasal	Forehead, upper eyelids, conjunctiva
Medial nasal	Nose, upper lip/philtrum, premaxilla, upper incisor teeth
Lateral nasal	Alae and base of the nose
Maxillary	Lower eyelids, cheeks, lateral parts of the upper lip, maxilla, canine, premolar and molar teeth
Mandibular	Whole lower lip, lower jaw (mandible, including teeth)

The facial processes fuse at different times; maxillary – 6 weeks, upper lip – 8 weeks and palate – 12 weeks (O’Rahilly, 1972; Danescu et al., 2015). Molecular studies have shown that the growth, structure and patternation of the facial primordia is controlled by a series of complex interactions that involves many factors such as fibroblast growth factors, sonic hedgehog proteins, bone morphogenetic proteins, homeobox genes *Barx1* and *Msx1*, the distal-less homeobox (*Dlx*) genes, and local retinoic acid gradients (Barlow et al., 1999; Hu and Helms, 1999; Lee et al., 2001; Ashique et al., 2002; Mossey et al., 2009; Marcucio et al., 2015; Parsons et al., 2015). The fusion between the facial processes depends on a series of events involving cell migration, growth, adhesion, differentiation and apoptosis. Disruptions in the fusion of the facial processes may result in complete or partial clefts of the face, lip and/or palate. The epithelial precursor periderm is involved in cellular adhesions with associated genes *IRF6, IKKA, SFN, RIPK4, CRHL3* all of which are under the transcription control of the transcription factor p*63* that influences the fusion process and differentiation of the epidermis (Hammond et al., 2017). For detailed embryological development the reader should read the original articles or illustrated reviews (Som and Naidich, 2013, 2014). Post-natally, facial growth tends to follow general somatic growth with periods of steady increments in size interspersed with periods of rapid growth with the peak growth occurring at puberty (Tanner et al., 1966a,b; Bhatia et al., 1979; Kau and Richmond, 2008; Richmond et al., 2009; Richmond S. et al., 2018). The timing, vectors and duration of surges in facial growth tend to be different for males and females and between populations contributing to overall facial variation (Kau et al., 2010; Hopman et al., 2014; Richmond R.C. et al., 2018).

## Acquiring Facial Surface Morphology and Describing/Quantifying Facial Shape

There are many imaging systems available to capture the external facial surface topography such as photography, lasers, photogrammetry, magnetic resonance Imaging (MRI), computerized tomography (CT), and cone beam computerized tomography (CBCT). Many of these techniques have been evaluated in terms of facial coverage, speed of capture, processing time, accuracy, validity and cost (Kovacs et al., 2007; Heike et al., 2010; Kuijpers et al., 2014; Tzou et al., 2014). For an individual who can sit still with a neutral facial posture in natural head position, the speed of capture is not critical. Even with relatively long acquisition times for some photogrammetric, MRI, CT, and CBCT systems, facial landmark reliability of less than 0.5 mm can be achieved (Kau et al., 2005, 2007; Liu et al., 2012). For infants and individuals with unpredictable facial or bodily movements a faster acquisition time will be required although reliability of achieving the same facial posture will be significantly reduced. Facial surfaces that are captured in supine and prone position often show slight differences (Munn and Stephan, 2018).

Assessment and judgment of the face and body can be traced back to the ancient Greeks and Egyptians when mathematical methods such as Fibonacci series and the golden proportion (1:1.618) were applied to art and architecture as a method of defining attractiveness and beauty (Ricketts, 1982). Facial features can be broadly characterized in terms of the size and shape of the whole face and/or its component parts (e.g., big/small head; short/long and wide/thin face, prominent or retrusive chin). Recognition of distinctive facial and lip features such as grooves, nodules, lip demarcation lines has also been reported (Merks et al., 2003; Wilson et al., 2013). To quantify facial features, landmarks have been traditionally used, taken either directly from the face or derived from photographs or radiographs. These landmarks are defined by identifiable/describable facial features, e.g., nasion, inner/outer canthi, commissures that can generate Euclidean distances, angles, and ratios (Farkas et al., 2002, 2004, 2005). One or more facial landmarks can be used to generate principal components, geodesic distances, geodesic arrays, facial shells and signatures which can categorize patterns in facial features (Hammond and Suttie, 2012; Hallgrimsson et al., 2015; Tsagkrasoulis et al., 2017; Abbas et al., 2018). In addition, anthropometric masks have been proposed whereby five landmarks are used to crudely orientate the 3D facial shells which are then non-rigidly mapped on to a template which generates about 10,000 quasi landmarks (Claes et al., 2012). Asymmetry is preserved in some of these techniques. However, if the facial shell is reflected on to the opposite side any facial asymmetry will be lost.

The various acquisition techniques (photographs, MRI, laser and photogrammetry) have been used in different studies and all have identified the *PAX3* gene associated with the shape of the nasal root area (Liu et al., 2012; Paternoster et al., 2012; Adhikari et al., 2016; Shaffer et al., 2016; Claes et al., 2018). The use of ordinal and quantitative measures has been explored reporting good correlation with inter-alae and lower lip distances (*r* = 0.7) and poor association for naso-labial angle (*r* = 0.16) (Adhikari et al., 2016).

## Disentangling Genetic and Environmental Factors

### Normal Facial Surface Morphology

Standardized clinical facial charts/tables/measures are routinely used for newborns (e.g., head circumference, body length) and other specialties such as, ophthalmology and orthodontics. There are many published norms for different racial/population groups used to identify individuals who fall within the normal range and identify any facial dysmorphologies.

The soft tissue facial variation has been explored in a large Caucasian population of 15-year-old children (2514 females and 2233 males) recruited from the Avon Longitudinal Study of Parents and Children (ALSPAC). Face height (28.8%), width of the eyes (10.4%) and prominence of the nose (6.7%) explained 46% of total facial variance (Toma et al., 2012). There were subtle differences between males and females in relation to the relative prominence of the lips, eyes, and nasal bridges including minor facial asymmetries (Toma et al., 2008, 2012; Wilson et al., 2013; Abbas et al., 2018). The dimorphic differences appear to follow similar patterns in different ethnic groups (Farnell et al., 2017).

### Heritability

Facial morphology refers to a series of many different complex traits, each influenced by genetic and environmental factors. In particular, the strong effects that genetic variation can have on facial appearance are highlighted by historical portraits of the European royal family, the Habsburgs (1438–1740). Presumably because of frequent consanguineous marriages, later Habsburg rulers often had extreme facial phenotypes such as the characteristic “Habsburg” jaw (mandibular prognathism). Indeed, the last Habsburg King of Spain, Charles II, was reported to have had difficulties eating and speaking because of facial deformities. The influence of genetic variation is also evident in non-consanguineous families, where dental and facial characteristics are common among siblings and passed on from parents to their offspring (Hughes et al., 2014). Twin studies have historically been employed to explore the relative genetic and environment influence on facial shape exploiting the genetic differences between monozygotic and dizygotic twins (Visscher et al., 2008). Twin studies suggest that 72–81% of the variation of height in boys and 65–86% in girls is due to genetic differences with the environment explaining 5–23% of the variation (Jelenkovic et al., 2011). Similar levels of genetic-environmental contributions have been reported for some facial features. Predominantly genetic influences have been reported for anterior face height, relative prominence of the maxilla and mandible, width of the face/nose, nasal root shape, naso-labial angle, allometry and centroid size (Carels et al., 2001; Carson, 2006; Jelenkovic et al., 2010; Djordjevic et al., 2013a,b, 2016; Cole et al., 2017; Tsagkrasoulis et al., 2017). Substantial heritability estimates for facial attractiveness and sexual dimorphism (0.50–0.70 and 0.40–0.50), respectively (Mitchem et al., 2014), further demonstrate the strong genetic influences on facial phenotypes.

Contrastingly, previous estimates suggest that antero-posterior face height, mandibular body length, ramus height, upper vermillion height, nasal width and maxillary protrusion are more strongly influenced by environmental factors (Jelenkovic et al., 2010; Djordjevic et al., 2016; Sidlauskas et al., 2016; Cole et al., 2017; Tsagkrasoulis et al., 2017). However, it is important to note that heritability estimates for specific traits can be inconsistent for a number of reasons including heterogeneity across study populations, small sample sizes, research designs, acquisition methods and the differing types of analyses employed.

### Environmental Influences

From the moment of conception, the parental environment can influence the development of the fetus. Facial development occurs very early at a time when the mother is not always aware that she is pregnant. The developing fetus may be subject to adverse environments at home, in the workplace or through lifestyle activities (smoking, alcohol and drug intake, allergens, paint, pest/weed control, heavy metals, cleaning, body products such as perfumes and creams). Many of these substances can cross the placenta (Naphthalene a volatile polycyclic aromatic hydrocarbon related to solvent emissions is present in household products and pesticides – Mirghani et al., 2015; Nicotine – Wickström, 2007; Drugs and alcohol – Lange et al., 2014). There is evidence to suggest that the effects of some of these substances can also continue post-natally through breast milk fed to the new-born (heavy metals – Caserta et al., 2013; Dioxin – Rivezzi et al., 2013). Some of these early factors such as nictotine and alcohol may potentially influence on early neurological development (Wickström, 2007). Indeed, there is evidence to suggest that high levels of prenatal alcohol exposure can influence facial morphology; individuals with fetal alcohol syndrome disorders can present with facial abnormalities (Hoyme et al., 2016) as well as other developmental anomalies such as caudate nucleus asymmetry and reduced mass of the brain (Suttie et al., 2018). However, the effects of lower levels of prenatal alcohol exposure on facial morphology are less clear (Mamluk et al., 2017; Muggli et al., 2017; Howe et al., 2018c). Similarly, it has been hypothesized that maternal smoking may influence facial morphology and be a risk factor for cleft lip and palate (Xuan et al., 2016) with DNA methylation a possible mediator (Armstrong et al., 2016). However, to date one study has indicated that maternal smoking may interact with the *GRID2* and *ELAVL2* genes resulting in cleft lip and palate (Beaty et al., 2013). However, previous studies investigating gene-smoking interactions in the etiology of birth defects have produced mixed results (Shi et al., 2008). Another mechanism via which environmental influences can affect facial traits is natural selection, where certain facial traits may have beneficial effects on reproductive fitness. For example, there is evidence that nose shape has been under historical selection in certain climates (Weiner, 1954; Zaidi et al., 2017).

Generally, most modifiable environmental factors have only subtle effects on the face. However, it is important to note that stochastic chance events such as facial trauma, infections, burns, tumors, irradiation and surgical procedures can all have a significant impact on facial development and consequently facial morphology.

## Craniofacial Shape Gene Discovery

The first wave of genetic studies of craniofacial Mendelian traits were based on linkage or candidate gene studies of genetic loci known to be involved in craniofacial development or genetic syndromes affecting the face. Down syndrome, cleft lip and/or palate, Prader-Willi syndrome, and Treacher Collins syndrome can all present with facial abnormalities and genetic loci associated with them have been studied in relation to normal facial development (Boehringer et al., 2011; Brinkley et al., 2016).

**FIGURE 1:**
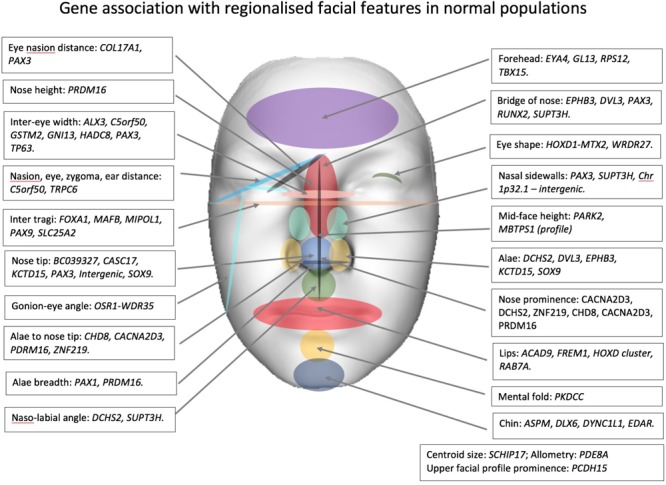
Gene association with regionalized facial features in normal populations.

Genome-wide association studies (GWAS) have investigated the association between normal facial variation and millions of single nucleotide polymorphisms (SNPs). GWAS studies coupled with high-resolution three-dimensional imaging of the face have enabled the study of the spatial relationship of facial landmarks in great detail. Over the last 6 years there has been significant progress with 9 published GWAS which have identified over 50 loci associated with facial traits (Liu et al., 2012; Paternoster et al., 2012; Adhikari et al., 2016; Cole et al., 2016; Shaffer et al., 2016; Lee et al., 2017; Cha et al., 2018; Claes et al., 2018; Crouch et al., 2018). The genes and broad regional associations are shown in **Table 2** (ordered by facial feature and chromosome) and **Figure 1** (showing facial region). For detailed information on the biological basis of individual genes, the reader should refer to the original articles. Different facial measures have been applied to facial images obtained from a variety of acquisition systems (2D and 3D). Genes are likely to influence more than one facial trait. For instance, the *PAX3* gene is associated with eye to nasion distance, prominence of the nasion and eye width, side walls of the nose, and prominence of nose tip. Similarly, the naso-labial angle will be associated with nose prominence and *DCHS2* is linked to both traits.

**Table 2:** List of genes and SNP’s associated with normal variation ranked by chromosome position (GWAS).

Facial feature	Facial phenotype	Chr position	SNP’s	Genes	Replication p values	Mutations	Function	Interactions	Author
Ala aperture	Subtle prominent area around alae and wider entry to nostrils.	4q31.3 (154828366)	rs9995821	*DCHS2*	*7.33 × 10^-19^*		Encodes a large protein that contains many cadherin domains and likely functions in cell adhesion		Claes et al., 2018
Allometry	Allometry (scaling size and shape), inner canthal distance, overall face shape and width.	15 (85029945), (85061095)	rs12908400, rs12909111	*PDE8A*	*1 × 10^-6^*		High expression in the ectoderm of the nasal, maxilla, and mandibular prominences. Transcriptional element observed in endothelial cells		Cole et al., 2016
Centroid size	Centroid size, face height and face width but independent of centroid size.	3q25.33	rs79909949	*SCHIP*	*1.8 × 10^-3^*		Predicted transcriptional regulatory element. An enhancer in many different cell types including an open hypomethylated chromatin configuration, multiple DNase I hypersensitivity sites, and numerous RNA polymerase II and transcription factor binding sites.		Cole et al., 2016
Chin prominence	Centralized prominence of chin extending to lower lip associated to mandibular recession in line with the commissures.	1q31.3 (197329041)	rs2821116	*ASPM*	*2.29 × 10^-15^*	Associated with microcephaly	Essential for normal mitotic spindle function in embryonic neuroblasts	Interacts with SOX9 through transcription factors	Claes et al., 2018
Chin prominence	Prominence of chin button associated with lateral recession.	7q21.3 (96124975)	rs10238953	*DLX6, DYNC1L1*	*1.29 × 10^-26^*		Homeobox transcription factor gene, encode proteins with roles in forebrain and craniofacial development		Claes et al., 2018
Chin prominence and shape	Chin shape and protrusion	2q12	rs3827760	*EDAR*	*4 × 10^-10^*	Hypohidrotic ectodermal dysplasia	Encodes a member of the tumor necrosis factor receptor family		Adhikari et al., 2016
Eye shape	Curvature of eyelid	2:176,820,065	rs970797	*HOXD1-MTX2*	*0.03*		Transcription factor that is involved in differentiation and limb development		Cha et al., 2018
Eye shape	Eye tail length	6:169,699,889	rs3736712	*WDR27*	*0.029*		May form scaffolds for protein-protein interaction and play key roles in cell signaling		Cha et al., 2018
Eye width	Distance between the eyeballs and nasion	2q35	rs974448	*PAX3*	*0.002*				Shaffer et al., 2016
Eye width	Distance between eyeballs	2q35 (222713558)	rs16863422 rs12694574 rs974448	*PAX3*	*1.6 × 10^-8^*	Waardenburg syndrome	Transcription factor expressed in neural crest cells, a multipotent cell population contributing to most differentiated cell types in the vertebrate face.		Liu et al., 2012
Eye width	Distance between eyeballs	3q28 (191032117)	rs17447439	*TP63*	*4.4 × 10^-8^*	Ankyloblepharon-ectodermal defects-cleft lip/palate (AEC) syndrome	Important roles in orchestrating developmental signaling and epithelial morphogenesis		Liu et al., 2012
Eye width	Inter canthi distance	Xq13.2 (72289467)	rs11093404	*PABP1- C1L2A, HADC8*	*4.16 × 10^-8^*	Cornelia de Lange syndrome, Facial dysmorphology and hypertelorism.	Encodes an uncharacterized poly-A binding protein, encodes a histone deacetylase involved in epigenetic gene silencing during craniofacial development		Shaffer et al., 2016
Eye width and depth	Eyes height width and depth		rs7560738	*TMEM163*		Mucolipidosis type IV (MLIV)	Member of the cadherin superfamily. Coding for a transmembrane protein that is a putative zinc transporter expressed in the brain and retina, as well as in a limited number of other tissues.		Crouch et al., 2018
Eye width and depth	Distance between eyeballs and nasion	10q24.3 (105800390)	rs805722	*COL17A1*	*5.9 × 10^-4^*	Junctional epidermolysis bullosa	May be involved in craniofacial patternation		Liu et al., 2012
Face height	Mid-face height	6q26	rs9456748	PARK2	5 × 10*^-^*^8^	Juvenile-onset Parkinson disease. Disk degeneration, cholesterol levels.	Encodes a protein involved in proteasomal degradation		Lee et al., 2017
Face height/depth	Right gonion to exR and enR	2:19,595,772	rs7567283	OSR1-WDR35	0.046	Sensenbrenner syndrome/cranio-ectodermal dysplasia	Broad expression in salivary gland/cell cycle progression, signal transduction, apoptosis		Cha et al., 2018
Face width	Inter tragi distance	14q21.1 (38038468)	rs17106852	*PAX9, SLC25A2, MIPOL1, FOXA1*	*1.01 × 10^-8^*	Holoprosencephaly	Dental and craniofacial development		Shaffer et al., 2016
Face width	Inter tragi distance	20q12 (38904203)	rs6129564	*MAFB*	*1.65 × 10^-9^*	Orofacial clefts, multicentric carpotarsal osteolysis syndrome	Migration of cranial neural crest cells and patternation of the craniofacial complex		Shaffer et al., 2016
Facial depth	Left tragus to nasion	11q22.1 (101394765)	rs12786942	*TRPC6,*	*4.5 × 10^-8^*		Encodes a cation channel subunit mutated in hereditary renal disease	Associated with Sox9	Shaffer et al., 2016
Forehead	Centralized prominence of forehead with vertical	1p12 (119762175)	rs72691108	*TBX15*	*1.01 × 10^-15^*	Cousin syndrome. Craniofacial,	Encodes a phylogenetically conserved family of transcription		Claes et al., 2018
	depression above the orbits					cervical, auricular, and skeletal malformation.	factors that regulate a variety of developmental processes		
Forehead	Recessive central portion of forehead with prominence laterally.	6q23.2 (133615646)	rs5880172	*RPS12, EYA4*	*6.91 × 10^-13^*	Post-lingual, progressive, autosomal dominant hearing loss at the deafness, autosomal dominant non-syndromic sensorineural 10 locus	This gene encodes a ribosomal protein that is a component of the 40S subunit/may act as a transcriptional activator through its protein phosphatase activity, and it may be important for eye development		Claes et al., 2018
Lip (upper)	Central upper lip height	9p22	rs72713618	FREM1	2 × 10^-8^	Manitoba oculotrichoanal syndrome/BNAR (bifid nose with or without anorectal and renal anomalies). Trigonocephaly.	Expression in the midline where the left and right medial nasal processes fuse. Failure of neighboring embryonic tissues to fuse properly due to impairment of the basement membranes’ anchoring function.		Lee et al., 2017
Lip prominence	Prominent lips, lateral retrusive to upper and lower lips; slight narrowing of nasolabial crease to nasal sidewalls with small prominence superiorly.	2q31.1 (177111819)	rs970797	*HOXD cluster*	*1.12 × 10^-3^*		morphogenesis in all multicellular organisms		Claes et al., 2018
Mental fold	Prominence of mental fold, with subtle retrusive effects on labio-mandibular crease.	2p21 (42181679)	rs6740960	*PKDCC*	*3.44 × 10^-6^*		Protein coding.		Claes et al., 2018
Nasion prominence	Prominence and vertical position of nasion	2q35	rs7559271	*PAX3*	*4 × 10^-7^*	Waardenburg syndrome, craniofacial-deafness-hand syndrome	Active in neural crest cells. These cells migrate from the developing spinal cord to specific regions in the embryo. The protein made from the PAX3 gene directs the activity of other genes that signal neural crest cells to form specialized tissues or cell types such as some nerve tissue and pigment-producing cells called melanocytes.		Paternoster et al., 2012
Nasion, eyes and zygoma prominence	Nasion position relative to zygoma and eyeballs	5q35.1 (171061069)	rs6555969	*C5orf50*	*7.5 × 10^-5^*	Preaxial polydactyly and holoprosencephaly (HPE), a defect in development of the forebrain and midface	Craniofacial patternation		Liu et al., 2012
Nose bridge	Small depression superior to tip of the nose, associated with small depressed areas in the middle of the nasal side walls. Increased prominence bridge of nose.	1p32.1 (60997570)	rs4916068	*intergenic*	*1.39 × 10^-12^*				Claes et al., 2018
Nose bridge	Associated with small transverse ridges across the bridge of the nose prominent midway and less prominent across the alae.	3q27.1 (184333169)	rs58022575	*EPHB3, DVL3*	*2.39 × 10^-9^*		Ephrin receptors and their ligands, the ephrins, mediate numerous developmental processes, particularly in the nervous system/encodes a cytoplasmic phosphoprotein that regulates cell proliferation		Claes et al., 2018
Nose prominence	Left ala to tip of nose	14q11.2 (21365801)	rs21365801	*ZNF219, CHD8,*	*3.36 × 10^-8^*	Associated with autism spectrum disorder in conjunction withmacrocephaly and distinct facial features including a broad nose	Encodes a transcriptional partner of Sox9		Shaffer et al., 2016
Nose prominence	Nasal labial angle and nose prominence	17:67,537,404	rs2193054	SOX9	4.60 × 10^-7^/1.93 × 10^-8^	Campomelic dysplasia often with sex reversal	Acts during chondrocyte differentiation and, with steroidogenic factor 1, regulates transcription of the anti-Muellerian hormone (AMH) gene		Cha et al., 2018
Nose prominence	Recessive tip of nose with increased width along the sidewall of the nose.	17q24.3 (69139583)	rs11655006	*BC039327/CASC17*	*1.77 × 10^-5^*		ncRNA		Claes et al., 2018
Nose prominence	Prominent superior to tip of nose associated with a localized area of recession just above the alae.	19q13.11 (34290995)	rs287104	*KCTD15*	*2.86 × 10^-5^*		Protein coding		Claes et al., 2018
Nose prominence	Columella inclination, nose protrusion, nose tip angle	4q31 (155235392)	rs12644248	*DCHS2*	*4 × 10^-3^*	Campomelic dysplasia	Calcium dependent cell-adhesion protein, regulatory controlling cartilage differentiation	SOX9	Adhikari et al., 2016
Nose prominence and width	Nose width, nose height and left and right alae to prn.	1p36.32	rs4648379	*PRDM16*	*1.70 × 10^-5^*		Protein coding	Associated with PAX3	Shaffer et al., 2016
Nose width	Prominent lateral tips of the nose associated with slightly reduced alar wings.	17q24.3 (70036479)	rs5821892	*SOX9*	*3.01 × 10^-4^*	Skeletal malformation syndrome campomelic dysplasia, frequently with sex reversal.	Acts during chondrocyte differentiation and, with steroidogenic factor 1, regulates transcription of the anti-Muellerian hormone (AMH) gene.	SOX9 Interacts with CASC17 through transcription	Claes et al., 2018
Nose width	Nose width and nose height	1p36.23-p33 (3251376)	rs4648379	*PRDM16*	*1 × 10^-8^*	NSCL/P cleft and other craniofacial defects including mandibular hypoplasia. Left ventricular non-compaction 8 (LVNC8)	Thought to act in downstream mediation of TGFb signaling in developing orofacial tissues		Liu et al., 2012
Nose width	Alae width	20:37,426,155	rs2206437	DHX35	8.67 × 10*^-^*^4^		Cellular growth and division/involved in the import of proteins into the mitochondrion		Cha et al., 2018
Nose width	Nose wing breadth	20p11 (22041577)	rs927833	*PAX1*	*4* × *10^-3^*	Oto-facio-cervical syndrome	Transcription factor affecting chondrocyte differentiation	RUNX2 and SOX9	Adhikari et al., 2016
Nose width	Inter alae width	20p11.22 (21632545)	rs2424399	*PAX1*	*2.62* × *10^-8^*	Otofaciocervical syndrome	Chondrocyte differentiation		Shaffer et al., 2016
Nose width	Prominence at nasion and tip/alae width if nose with reduced width along the sidewalls of the nose.	2q36.1 (223039052)	rs10176525	*PAX3*	*4.39* × *10^-11^*	Waardenburg syndrome, craniofacial-deafness-hand syndrome, and alveolar rhabdomyosarcoma	Critical roles during fetal development		Claes et al., 2018
Nose width	Nose bridge breadth	6p21 (45329656)	rs1852985	*SUPT3H/RUNX2*	*5* × *10^-3^*	Cleidocranial dysplasia	Bone development		Adhikari et al., 2016
Eye width	Intercanthal width	1p13.3 (110218761)	rs619686	*GSTM2, GNAI3, ALX3*	*4.7 × 10^-8^*	Auriculocondylar syndrome	Pharyngeal arch patterning		Shaffer et al., 2016
Nose width	Depression nasion associated with wider of nasal side walls superiorly positioned. Also slight depression at subnasale.	6p21.1 (44681840)	rs227833	*SUPT3H*	*5.63* × *10^-4^*		Protein coding.		Claes et al., 2018
Nose width	Nose wing breadth	7p13 (42131390)	rs17640804	*GL13*	*6* × *10^-3^*	Greig cephalopolysyndactyly syndrome	Activator and repressor in the sonic hedgehog signaling pathway	GLI3 and PAX1 (nose wing)	Adhikari et al., 2016
Philtrum	Depression of philtrum with prominent philtral pillars; reduced ale and prominent subnasale.	3q21.3 (128106267)	rs2977562	*RAB7A, ACAD9*	*8.63* × *10^-7^*	Charcot-Marie-Tooth (CMT) type 2 neuropathies	Regulates vesicle traffic in the late endosomes and also from late endosomes to lysosomes.		Claes et al., 2018
Upper facial profile height	Skeletal pattern/mandibular profile		rs11642644	*MBTPS1*	2.7 × 10*^-^*^3^		Involved in craniofacial patternation.		Crouch et al., 2018
Upper facial profile prominence	Skeletal pattern/mandibular profile		rs2045145	*PCDH15*	2.85 × 10^-3^	Usher syndrome, which involves hearing and balance malfunction in addition to retinal defects.			Crouch et al., 2018

Some reported genes appear to influence different parts of the face. *PRDM16* is linked to the length and the prominence of the nose as well as the width of the alae, *SOX9* is thought to be related to the shape of the ala and nose tip, variation in *SUPT3H* is thought to influence naso-labial angle and shape of the bridge of the nose, while centroid size (squared root of the squared distances of all landmarks of the face from the centroid) and allometry (relationship of size to shape) have been linked to *PDE8A* and *SCHIP17* genes, respectively, (Cole et al., 2016). Eye width and ear – nasion distance and nasion -zygoma – eyes distances are linked to *C5orf50*. There is some evidence to suggest that there are additive genetic effects on nose shape involving *SOX9, DCHS2, CASC17, PAX1, RUNX2*, and *GL13* and chin shape, *SOX9* and *ASPM*. In addition, it is likely that one or more genes influence the whole shape of the face as well as more localized facial regions (Claes et al., 2018). A significant number of genes are integrally involved in cranial neural crest cells and patternation of the craniofacial complex (e.g., *C5orf50, MAFB*, and *PAX3*).

## Understanding the Etiology of Craniofacial Anomalies

Identifying genetic variants influencing facial phenotypes can lead to improved etiological understanding of craniofacial anomalies, advances in forensic prediction using DNA and testing of evolutionary hypotheses.

Non-syndromic cleft lip/palate (nsCL/P) is a birth defect with a complex etiology, primarily affecting the upper lip and palate (Mossey et al., 2009; Dixon et al., 2011). Previous studies have identified genes associated with both nsCL/P and facial phenotypes; such as variation in *MAFB* which is associated with face width in normal variation (Beaty et al., 2010, 2013; Boehringer et al., 2011; Liu et al., 2012; Peng et al., 2013; Shaffer et al., 2016). Furthermore, craniofacial sub-phenotypes have been observed in nsCL/P cases and their unaffected family members such as orbicularis oris muscle defects and facial shape differences supporting the existence of nsCL/P related sub-phenotypes (Stanier and Moore, 2004; Marazita, 2007; Neiswanger et al., 2007; Menezes and Vieira, 2008; Weinberg et al., 2009; Aspinall et al., 2014).

The important link between facial variation and nsCL/P is highlighted by a study comparing facial morphologies (linked to genes) of children with nsCL/P and unaffected relatives. There was reduced facial convexity (*SNAI1*), obtuse nasolabial angles, more protrusive chins (*SNAI1, IRF6, MSX1, MAFB*), increased lower facial heights (*SNAI1*), thinner and more retrusive lips and more protrusive foreheads (*ABCA4-ARHGAP29, MAFB*) in the nsCL/P relatives compared to controls. There was also greater asymmetry in the nsCL/P group (*LEFTY1, LEFTY2, and SNAI1*) (Miller et al., 2014).

There is evidence that nsCL/P genetic risk variants have an additive effect on philtrum width across the general population. This association suggests that developmental processes relating to normal-variation in philtrum development are also etiologically relevant to nsCL/P, highlighting the shared genetic influences on normal-range facial variation and a cranio-facial anomaly (Howe et al., 2018a).

Similarly, genetic variations associated with normal-range facial differences have been linked to genes involved in Mendelian syndromes such as *TBX15* (Cousin syndrome) (Shaffer et al., 2017; Claes et al., 2018), *PAX1* (Otofaciocervical syndrome) (Shaffer et al., 2016) and *PAX3* (Waardenburg syndrome) (Paternoster et al., 2012). It has been hypothesized that deleterious coding variants may directly cause congenital anomalies while non-coding variants in the same genes influence normal-range facial variation via gene expression pathways (Shaffer et al., 2017; Freund et al., 2018).

Shared genetic pathways may influence both normal-range variation in facial morphology and craniofacial anomalies. Disentangling these shared pathways can improve understanding of the biological processes that are important during embryonic development.

### Estimating Identity

#### Anthropology and Human History

Over time, facial morphology across populations has been influenced by various factors, such as migration, mate-choice, survival and climate, which have contributed to variation in facial phenotypes. Genetic and facial phenotype data can be used to improve understanding of human history.

#### Ancestry and Genetic Admixture

Ancestry and physical appearance are highly related; it is often possible to infer an individual’s recent ancestry based on physically observable features such as facial structure and skin color. Indeed, previous studies have demonstrated that self-perceived and genetically inferred ancestry are associated with facial morphology, particularly with regards to the shape of the nose (Dawei et al., 1997; Le et al., 2002; Farkas et al., 2005; Claes et al., 2014). Facial morphological differences relating to ancestry are well-characterized when comparing individuals from distinct populations, but distinct differences remain even within more ancestrally homogeneous populations.

Historical migrations, such as the European colonization of Latin America, led to genetic admixture (breeding between individuals from previously isolated populations) (Hellenthal et al., 2014), which greatly influenced the facial morphology of the Latin American population. Indeed, modern day Latin Americans have mixed African, European and Native American ancestry, with genetic admixture highly predictive of physical appearance. For this reason, ancestral markers are often included in facial prediction models (Claes et al., 2014; Ruiz-Linares et al., 2014; Lippert et al., 2017).

#### Mate Choice, Sexual Dimorphism and Selection

Facial phenotypes can influence mate choice and be under selection pressures. These factors can then affect reproductive behavior and lead to population-level changes in facial variation as certain facial phenotypes are favored. Previous studies have suggested that facial features such as attractiveness (Little et al., 2001; Fink and Penton-Voak, 2002), hair color (Wilde et al., 2014; Adhikari et al., 2016; Field et al., 2016; Hysi et al., 2018), eye color (Little et al., 2003; Wilde et al., 2014; Field et al., 2016) and skin pigmentation (Jablonski and Chaplin, 2000, 2010; Wilde et al., 2014; Field et al., 2016) may influence mate choice and/or have been under historical selection. Features related to appearance are also often sexually dimorphic, possibly as a result of sexual and natural selection. For example, significantly more women self-report having blonde and red hair while more men as self-report as having black hair (Hysi et al., 2018).

The possible evolutionary advantages of facial phenotypes have been discussed extensively but anthropological hypotheses can be tested using genetic and facial phenotype data. For example, a masculine face has been hypothesized to be a predictor of immunocompetence (Scott et al., 2013). A previous study tested this hypothesis using 3D facial images and genetic variation in the major histocompatibility complex (MHC) region and found weak evidence to support this (Zaidi et al., 2018). Other possible benefits that have been explored include: the fitness advantages of hair color (Adhikari et al., 2016; Hysi et al., 2018), nasal shape and climate adaptation (Zaidi et al., 2017) and the benefits of darker skin pigmentation (Wilde et al., 2014; Aelion et al., 2016). Strong levels of phenotypic and genotypic spousal assortment have been previously demonstrated for height (Robinson et al., 2017) and similar methods could be applied using facial phenotypes to explore the influences of facial morphology on mate choice.

#### The Use of Reverse Genetics for Forensic Prediction of Facial Features

The premise of reverse genetics is that there is known function of a gene or a group of genes which will create a particular phenotype with a degree of certainty. This has been proposed as a method to build a profile of facial features from a sample of DNA (Claes et al., 2014) but could also be used to determine previous health history or future health risks (Idemyor, 2014). This approach may be appropriate for unique facial characteristics but is more challenging when one or more genes are associated with the variation of facial phenotype (e.g., prominence of the nasal bridge or length of the nose, hair and eye color/tones). The *PAX3* gene is associated with the distance between the mid-endocanthion point and surface nasion with a mean distance of 17.5 mm with differing axis values up to 6.7 mm (x), 17.7 mm (y), and 18.9 mm (z). Although, it is known that the *PAX3* influences the prominence of the bridge of the nose it is more challenging to know to what extent this influences adjacent facial regions in each individual. In addition, genetic and environmental factors will have subtle influences on the face. Although the molecular understanding of genetic variation influencing facial morphology is improving, the use of DNA as a prediction tool is still a long way off. However, recent studies suggest that DNA has the potential to identify an individual from a small group of possible candidates (Claes et al., 2014; Biedermann et al., 2015; Kayser, 2015). There is clearly a place in forensic science to develop a robust diagnostic tool to determine age, ancestry, appearance, relatedness and sex from DNA samples. One study effectively predicted eye color (85% for brown and 70% for blue), hair color (72% for brown, 63% for blonde, 58% for black, and 48% for red) and ancestry (56%); which are relatively low levels and individually could not be relied on for certain identifications but has greater potential when used collectively (Keating et al., 2013). The prediction of skin color from DNA has also been reported (Chaitanya et al., 2018) and DNA methylation has been demonstrated as a useful predictor of age. Age prediction using methylation techniques have indicated a mean absolute deviation of 5–8 years (Xu et al., 2015; Bocklandt et al., 2011; Hamano et al., 2017)

The determination of facial appearance, health history and future health risk from DNA is has great potential (Claes et al., 2014; Kayser, 2015; Toom et al., 2016) but caution should be expressed with respect to assumptions, interpretation and individual confidentiality as there is a significant threat to an individual in obtaining healthcare insurance (Hallgrimsson et al., 2014; Idemyor, 2014; Toom et al., 2016).

Previously published studies that have identified gene-phenotype associations which provides evidence of associations for complex facial traits which can be integrated into prediction models. The collective use of these techniques to identify the various facial features will increase the robustness of linking the DNA to a likely suspect/candidate.

## Shared Influences of Facial and Other Traits

**Table 2** highlights that genetic variants influencing facial morphology can have pleiotropic effects on parts of the body independent to the brain and surrounding craniofacial structures (e.g., cardiovascular, endocrine, gastro-intestinal, central nervous, musculo-skeletal and uro-genital systems). The growing number of GWAS datasets has allowed exploration of the shared genetic influences on different phenotypes (Bulik-Sullivan B. et al., 2015; Pickrell et al., 2016). There is the potential for relationships between medical and facial conditions to be explored using genetic summary data. The limited evidence for genetic correlation between facial and other traits has been reported in **Table 3**. It is important to note that the strong association between facial morphology and ancestry means that any correlations may be attributable to fine-scale population substructure.

**Table 3:** Reported shared influences of medical conditions, normal facial variation with associated genes.

Phenotype	Facial feature associated with medical conditions	Genes identified
Asymmetry	Facial asymmetry was more prevalent in 15-year-old ALSPAC children who had experienced an ear infection compared to a control group (Pound et al., 2014). There are numerous possible causes of mandibular asymmetry reported (Pirttiniemi, 1994) and middle ear infection is one (Güven, 2008). Asymmetry of the nasal root, nose tip and columella base has been associated with fasting insulin and Low-density Lipoproteins (Djordjevic et al., 2013b). Facial asymmetry has also been associated with neurological conditions explained by the close relationship during embryological development (Hennessy et al., 2004; Suttie et al., 2018).	
Face height	Juvenile-onset Parkinson disease. Face height, standing height, infant head circumference, length and weight at birth are closely related (Van der Beek et al., 1996; Mellion et al., 2013; Bulik-Sullivan B. et al., 2015). There are strong associations with onset of puberty, bone density, breast size, high and low-density lipoproteins (HDL, LDL) as well as overall cholesterol and BMI (Cousminer et al., 2013, 2014; Bulik-Sullivan B. et al., 2015; Pickrell et al., 2016). Delays in puberty will result in shorter stature and smaller face height (Verdonck et al., 1999). A series of case-control studies undertaken in the ALSPAC 15-year-old cohort investigating asthma, atopy and sleep disorder breathing (Al Ali et al., 2014a,b, 2015) found relatively small mixed opposite effects on face height.	*PARK2* with mid-face height.
Face width	Five genes have been reported for inter-tragi and seven genes for inter-eye distances. Association of face width and timing of tooth eruption and height has been reported (Fatemifar et al., 2013).	*FOXA1, MAFB, MIPOL1, PAX9, SLC25A2, ALX3, C5orf50, GSTM2, GNI13, HADC8, PAX3, TP63, MAFBHMGA2, AJUBA, ADK.*
Nose length/prominence	Nose prominence will be related to nose length evidenced by the reporting of *PRDM16* for both phenotypes. Mixed findings in relation to nose length have been reported for schizophrenia (Hennessy et al., 2010) and reported psychotic like symptoms in the 15-year-old ALSPAC cohort (Farrell, 2011). Schizophrenia has been associated with allergies, height, Crohn’s disease, Parkinson’s disease, HDL and near sightedness. Nose length has also been associated with LDL, total cholesterol and triglycerides (Pickrell et al., 2016).	*PRDM16, BC039327/ CAC17 DCHS2, ZNF219, CHD8, PRDM16*, SOX9 *SLC39A8APOE*
Nose width	12 genes have been reported for nose width. *SUPT3H* has been associated with height in the Korean population (Kim et al., 2010). Inter-ala distance wider in asthmatics (Al Ali et al., 2014b).	*PAX1, PRDM16, SOX9, GSTM2/GNAI3/ALX3, DHX35, PAX1, PAX, SUPT3H/RUNX2, GLI3*
Philtrum	Age of menarche Associated with *RAB7A*	*RAB7A*
Prominence of chin	Morphology of the chin has been linked with four genes. The mandible was less prominent in sleep disorder breathing (Al Ali et al., 2015).	*ASPM, DLX6, DYNC1L1, EDAR*

## Discussion

Normal facial development is dependent on Cranial Neural Crest Cells and correctly spatially positioned and differentiated tissues and structures that influence the shape and morphological features of the face. The disruption of neuro-facial developmental and maturational processes can lead to widespread and long-lasting abnormalities in central nervous system structure and functions and some of these disturbances will also be accompanied with subtle differences in facial features (Hennessy et al., 2010).

### Epigenetics

Epigenetics refers to mitotically (and perhaps, controversially meiotically) heritable changes in gene expression which are not explained by changes to the DNA base-pair sequence. Epigenetic processes include DNA methylation, histone modification and chromatin remodeling, which can affect gene expression by regulating transcription (Jaenisch and Bird, 2003; Bird, 2007; Gibney and Nolan, 2010; Allis and Jenuwein, 2016). Epigenetic processes are particularly relevant to craniofacial phenotypes because of the general importance of epigenetic gene regulation during embryonic development (Reik, 2007) and their specific role in neural crest development (Hu et al., 2014).

Craniofacial epigenetic studies to date have largely focused on orofacial clefts. Previous epigenome-wide association studies (EWAS) have found evidence of differential DNA methylation between cleft cases and controls (Alvizi et al., 2017), as well as between the different orofacial cleft subtypes (Sharp et al., 2017) implicating the relevance of DNA methylation in craniofacial development. The modifiable nature of epigenetic processes has led to much excitement that these processes may mediate the effect of environmental exposures. The maternal environment is thought to play an important role with regards to orofacial clefts. Previous studies have found strong evidence supporting associations between prenatal smoke exposure (Joubert et al., 2016) and folate supplementation (Richmond R.C. et al., 2018) with differential DNA methylation, but contrastingly there is no clear evidence for an association between prenatal alcohol exposure and DNA methylation (Sharp et al., 2018). In cleft lip tissue, limited evidence was found for an association between LINE-1 methylation and maternal exposures but conclusions were limited by modest sample sizes (Khan et al., 2018). Future work could utilize meditation techniques (Tobi et al., 2018) or Mendelian randomization (Relton and Davey Smith, 2012) to formally investigate the possibility that prenatal exposures influence orofacial cleft risk via epigenetic processes.

Similarly, epigenetic processes may mediate the effects of germline genetic variation. Many of the previously discussed genetic variants associated with facial traits in GWAS reside in non-protein coding regions of the genome with unclear functional relevance. One possibility is that these variants may influence facial phenotypes through gene regulation pathways involving epigenetic processes. Indeed, a previous study demonstrated that a major risk locus for non-syndromic cleft lip/palate (nsCL/P), in a non-coding interval, is involved in the regulation of gene expression in the developing murine face (Uslu et al., 2014) while another study found some evidence that nsCL/P genetic variants may influence nsCL/P risk via changes in DNA methylation and gene expression (Howe et al., 2018b).

Despite the promise of early craniofacial epigenetic studies, there are important caveats worth noting. First, a major issue is that epigenetic modifications can vary across different tissues. Previous studies have used DNA methylation in blood as a proxy for methylation in lip and palate tissues. Despite some evidence for positive correlation between blood and lip tissue DNA methylation (Alvizi et al., 2017; Howe et al., 2018b), the extent to which blood is a suitable proxy is unknown. Furthermore, it is unclear whether the epigenetic profile of lip and palate tissues postnatally are comparable to the same tissues during embryonic development. A previous orofacial cleft GWAS found no clear evidence for enrichment of tissue-specific signals, concluding that this may be attributable to a lack of suitable tissue types (Leslie et al., 2017). Second, when testing causality, epigenetic modifications can vary across the life-course, so it can be difficult to discern the direction of effect between an epigenetic modification and the phenotype. It is therefore important to use causal inference techniques such as epigenetic Mendelian randomization Relton and Davey Smith, 2012) or the Steiger test (Hemani et al., 2017) to orientate the likely directions of effect between phenotypes, epigenetic modifications and gene expression.

### Genome Regulatory Systems

The gene regulatory systems are complex and numerous and detailing these regulatory mechanisms has been the goal of the NIH Roadmap Epigenomics Project whereby next generation sequencing technologies (e.g., ChiP seq) are employed to map DNA methylation, histone modifications, chromatin accessibility in a variety of research media such as, animal models (mouse, chicken, zebrafish, frog, and primates) and stem cells and regulated human fetal tissues (Hochheiser et al., 2011; Roosenboom et al., 2016; Van Otterloo et al., 2016). Enhancers have a specific role in the expression of a target gene in different cells, anatomical regions and during different developmental time-points (Visel et al., 2009; Attanasio et al., 2013; Wilderman et al., 2018). The role of enhancers modifying histones, chromatin states are key for normal range craniofacial development and relative position of the various craniofacial tissues. Key transcriptional factors (activators or repressors) have been identified indicating extensive activation during early craniofacial development. These transcriptional factors may be limited to detail the precise facial shape or can be quickly activated in rapid periods of growth and development. Craniofacial enhancers have also been identified acting between the non-coding regions and proposed as a possible instrumental factor in some cleft cases (Wilderman et al., 2018).

### Complexity of Facial Features

The craniofacial region is made up of a series of complex structures which contribute to overall facial shape. Twin studies have indicated that facial shape is mainly due to genetic influences (≈75%) although the percentage variance explained in GWAS studies is extremely low generally explaining less than 2% of the total variance. GWAS may be underestimating and twin and family studies overestimating the levels of heritability. Facial shape and features are the result of mutations, genetic drift, recombination and natural selection. Rare Mendelian mutations, low frequency segregating variants, copy number variants and common variants contribute to complex phenotypes. Genetic interactions or epistasis may also explain the low levels of variance recorded. In addition, there is evidence of pleiotropy, quantitative phenotypes and Mendelian traits all influencing multiple phenotypes suggesting a large number of loci contribute additively to facial variation. Epigenetics focuses on the functional components of the genes and gene activities. The genome is comprised of 3.2 billion nucleotides wrapped in octomeric units of histones (chromatin). Modifications to chromatin through methylation, acetylation, phosphorylation or other processes are known to influence gene expression. Most epigenetic changes are transient and not generally heritable. However, a small proportion of epigenetic changes are transgenerational (Rachdaoui and Sarkar, 2014). However, there is a limited amount of evidence that epigenetic inheritance may carry over multiple generations (Schmidt and Kornfeld, 2016; Gluckman et al., 2007).

### Study Design, Sample Size and Power

Increasing the sample sizes of genetic studies of facial morphology through international collaborations, such as the type II diabetes consortia DIAGRAM (Morris et al., 2012), will help to improve the understanding of genetic associations and shared influences on facial traits (Evans, 2018). The availability of summary statistics on large GWAS studies will also enable the application of quantitative genetics methods to further investigate the genetic architecture of facial morphology. Polygenic risk scores, LD score regression (to reduce confounding biases Bulik-Sullivan B.K. et al., 2015) and Mendelian randomization can provide information on the genetic overlap of facial phenotypes with other genetic traits and the possibility to causally assess the association of risk factors with face development (Smith and Ebrahim, 2003).

Focusing on specific phenotypes and genetic variants in families will identify additional rare variants should be followed-up with a combination of genotyping and deep re-sequencing of the variants or genes of interest in large numbers of cases and controls. The analytical techniques (particularly the bootstrapped response-based imputation modeling (BRIM) and hierarchical spectral clustering analysis) employed by Claes et al. (2014, 2018) provide efficient and valid analyses and arguably more importantly, visual linkages between genetic variants and global shape. In addition, the individual facial traits have yielded impressive levels of significance using a relatively small number of subjects (Evans, 2018). Permutation testing is a valid alternative for more conservative tests such as Bonferroni (Sham and Purcell, 2014). The use of machine-learning and artificial intelligence approaches will be crucial in future GWAS studies to determine patterns and linkages in the numerous large data sets generated and archived related to craniofacial development functional genomics.

There have been nine GWAS studies and it is appropriate to try and integrate their findings through a meta-analysis. Different genetic models, genotyping and imputation techniques have been employed and the between-study heterogeneity should be considered.

### Phenotyping

Defining facial shape can be undertaken in different ways but it is important to appreciate that there will be associations with not only with other facial features but also body phenotypes and medical conditions. Visualizing and automatic detection of facial phenotypes and determining their prevalence in population groups will facilitate case-control evaluations to determine genetic variants. So far, all GWAS studies have studied the static face but capturing the face during simple facial actions in a population (dynamic movement with or without speech) will enable the exploration of combined neurological and morphological features by assessing both speed and range of movement.

### Population Cohorts

Population cohort studies enables researchers to study the environmental, disease and metabolic risk factors and genetic interactions from pre-birth throughout the lifecourse. Ideally facial images should be captured at birth, 5, 9, 12, 15, and 18 years of age and repeated every 10 years of age to capture facial features. During the pubertal growth period (9–18 years) facial images should be captured more frequently and if studying pubertal influences facial images should be captured at least every 6 months. Acquiring as much information as possible in relation known genetic additive effects, environmental factors and previous medical histories of family members (Grandparents, parents and offspring) will provide further insights into facial relatedness.

### Developing Prevention and Healthcare Strategies

With improving knowledge of the controlling mechanisms for normal and abnormal facial development, it is logical to pursue healthcare strategies in the first instance to prevent craniofacial anomalies arising, with discussion of risks with genetic counseling, possibly future gene therapies and the follow up with minimally invasive or non-surgical, scarless procedures to correct craniofacial anomalies such as cleft lip and palate and control vertical and horizontal growth particularly of the upper and lower jaws and nose. Surgical procedures are not always simple as often in CL/P patients there is often insufficient tissues available (epidermis/dermis, cartilage and bone with disrupted orientation of muscle fibers). Prevention may be challenging (other than continually improving environmental conditions and reducing exposure to potential epigenetic factors) as facial development occurs very early in gestation during a period whereby the mother is often unaware she is pregnant. Controlling the mechanisms of normal growth *in vivo* or alternatively *in vitro* creating similar morphological tissues with intact innervation, blood and lymphatic systems that could be transplanted later may become reality in the future. Future, environmental epigenetic studies will show whether particular chemicals map to corresponding sensitive genomic regions. It is important to identify early life exposures (particularly conception to birth) that may influence later life health outcomes.

## Conclusion

The face develops very early in gestation and facial development is closely related to the cranial neural crest cells. Disruption in early embryological development can lead to wide-ranging effects from subtle neurologic and facial features, which includes asymmetry, to significant impact on facial shape as characterized by a CL/P or in anomalies observed in craniofacial syndromes.

Heritability studies have provided insight into the possible genetic and environmental contributions to face shape. However, the sample sizes and inconsistencies in research design and particularly statistical management have yielded mixed results. Further detail is required on the heritability of facial features with particular attention to inherited pathways of specific facial features in homogenous populations and populations with significant admixture.

From birth to adulthood there are significant body and facial changes. Further work is required to explore the importance of the various biomedical markers and medical conditions (e.g., fasting glucose, cholesterol, asthma, and neurological disorders etc.) on the growth of the face, for example, remodeling of the facial skeleton, spatial changes of the constituent parts of the facial skeleton through sutures, condylar and nasal cartilages as well as the soft tissues, neural and vascular networks. The GWAS studies have provided insights into the genetic influences on facial shape. However, large-scale population studies are needed to identify more genetic variants not only in the context of facial shape but general body development with particularly attention to puberty. With any change in face shape the complex processes and communications at the biological and genome levels need to be identified and explained. The sheer volume of data collected in imaging genetics from images (hundreds of thousands of points), omics datasets (genomics, transcriptomics and cell-specific expression signals etc. – hundreds of millions of sequences) as well as biomarkers for medical conditions generates massive and complex data sets.

The size and heterogeneity of these data sets pose new challenges to efficiently and effectively, store, simplify and analyze the relative interactions and influences for a large number of face shape variables. The aim will be to continually develop and advance existing computerized tools and algorithms to solve these complex problems and this will require a multidisciplinary and internationally based team.

Impressions of an individual’s health are integral to social interactions and judgments are made on the visual appearance of skin, degree of roundness of the face and facial expression (Henderson et al., 2016). There has been significant progress in the first 6 years of GWAS and facial genetics. With increased sample sizes, improved understanding of shared genetic influences on human traits and advancement in techniques there is likely to be significant further progress in the next 6 years. Understanding the face will explain “why we look the way we do” a range of normality and abnormality that will be useful in healthcare applications and forensic science.

## Author Contributions

SR and LH outlined the overall manuscript. SR and SL wrote the section “Heritability”. LH and AZ wrote the section “Environmental Influences”. SR, ES, SL, and LH wrote the section “Craniofacial Shape Gene Discovery”. LH and SR wrote the section “Estimating Identity”. SR, ES, LH, and SL highlighted the shared facial traits. All authors actively participated in editing of the manuscript.

## Conflict of Interest Statement

The authors declare that the research was conducted in the absence of any commercial or financial relationships that could be construed as a potential conflict of interest. The handling Editor is currently collaborating with author SR and confirms the absence of any other collaboration.
